# Reconstruction techniques for cardiac cine MRI

**DOI:** 10.1186/s13244-019-0754-2

**Published:** 2019-09-23

**Authors:** Rosa-María Menchón-Lara, Federico Simmross-Wattenberg, Pablo Casaseca-de-la-Higuera, Marcos Martín-Fernández, Carlos Alberola-López

**Affiliations:** Laboratorio de Procesado de Imagen. Escuela Técnica Superior de Ingenieros de Telecomunicación, Universidad de Valladolid, Campus Miguel Delibes, Valladolid, 47011 Spain

**Keywords:** Review, Cine cardiac MRI, Medical image processing, MRI reconstruction, Cardiovascular diseases

## Abstract

The present survey describes the state-of-the-art techniques for dynamic cardiac magnetic resonance image reconstruction. Additionally, clinical relevance, main challenges, and future trends of this image modality are outlined. Thus, this paper aims to provide a general vision about cine MRI as the standard procedure in functional evaluation of the heart, focusing on technical methodologies.

## Key points

- Cardiovascular diseases remain the first cause of death, morbidity, and disability worldwide.
- Cine MRI is the standard image modality for cardiac function evaluation.
- Cardiac MRI is a hot topic with prospects of continuing to grow.
- Review of the state-of-the-art reconstruction techniques for dynamic cardiac MRI.

## Introduction

**Fig. 1:**
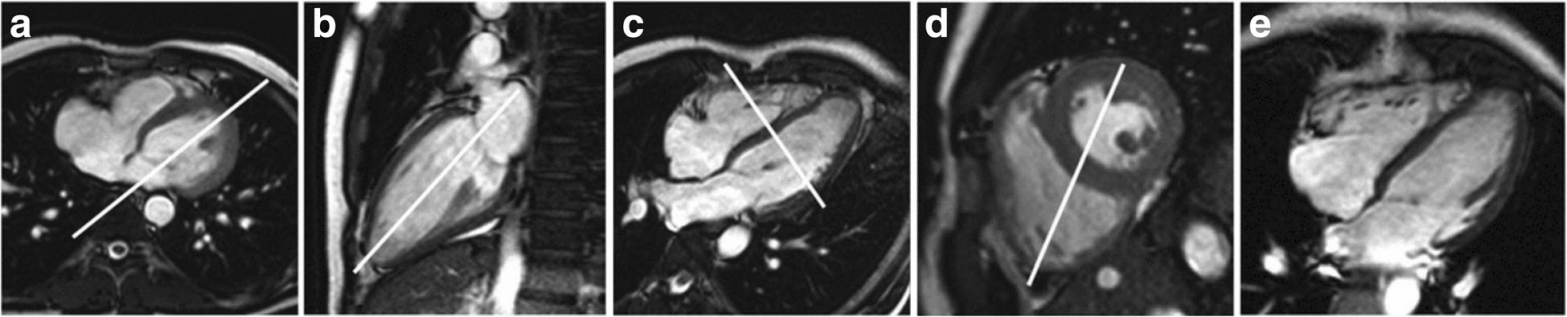
Cardiac MRI planes [7]. **a** Axial plane. **b** Vertical long-axis plane. **c** Horizontal long-axis plane. **d** Short-axis plane. **e** Four-chamber plane

Magnetic resonance imaging (MRI) has undeniably involved a revolution in medicine [1]. MRI is simultaneously a well-established and evolving area of cardiovascular medical imaging [2]. Diagnosis of cardiac diseases requires accurate assessment of function and morphology of the heart [3]. Cardiac MRI (CMRI) satisfies these requirements. Several features make CMRI a reference standard for the practice of cardiology. Its advantages are, among others, versatility, high reproducibility, and accuracy, which are unmatched by any other individual imaging modality [4]. CMRI is completely non-invasive, and it does not use ionizing radiation. Moreover, it provides high spatial resolution, wide field-of-view, and good soft tissue contrast [5, 6]. Furthermore, CMRI can provide a complete cardiovascular assessment of a patient in a single setting. Figure 1 illustrates the standard cardiac MRI planes used commonly in clinical practice to visualize the anatomy of the heart.

However, despite the aforementioned advantages, CMRI is still not a first-line study [1, 2]. It is often obtained when unanswered questions persist after other studies, such as echocardiography, radionuclide imaging, angiocardiography, or cardiothoracic CT [6]. This is owing to the expense of MRI technology, the lack of widespread availability, the absence of trained staff, the unfamiliarity of clinicians, and patient compliance. Note that CMRI is not always the most appropriate study for some patients. As an example, claustrophobic, uncooperative, and pediatric patients hinder the CMRI examination. In many cases, the administration of some kind of sedation is needed. Moreover, quality of MRI may be degraded due to artifacts induced by some kinds of metallic implants and foreign devices [4]. In particular, MRI is contraindicated in patients with certain aneurysm clips, cochlear implants, cardiac pacemakers, and cardioverter-defibrillator devices [4, 6]. However, CMRI is completely safe in patients with prosthetic cardiac valves or coronary stents.

The abovementioned positive factors, coupled with the high prevalence of cardiovascular diseases (CVDs) around the world [8], make CMRI a hot topic for both medical and technological research areas with prospects of continuing to grow. Taking this into account, the goal of this paper is to remark the key aspects, main challenges, and future trends on CMRI. More specifically, we focus on cine CMRI with a particular interest in fast acquisition and reconstruction procedures. In this sense, real-time cine imaging deserves a special mention because of its exceptional requirements for very fast reconstruction. With the present document, the authors aim to provide the reader with a general outlook of the state-of-the-art techniques in the field of cine CMRI.

### CMRI modalities

There are several modalities of CMRI with particular properties and applications. CMRI is used for the evaluation of many cardiac disorders: congenital heart diseases, cardiomyopathies, myocardial disease, cardiac masses and tumors, vascular diseases, and valvular and pericardial heart diseases, among others. A brief overview on MRI modalities used in cardiology is introduced below. For a detailed description of specific clinical indications, see references [1, 9].

Dynamic image sequences (cine) are required to acquire a complete information of the heart function throughout the cardiac cycle [2]. In fact, cine imaging is the most common technique in CMRI, and it is considered the gold standard for cardiac function evaluation [1]. Cine CMRI is especially useful for quantifying global and regional left and right ventricular function by measuring parameters such as stroke volume, ejection fraction, end-diastolic and end-systolic volumes, and masses [1, 3, 5].

Coronary MR angiography (MRA) is a promising imaging technique for detection of coronary artery disease (CAD). MRA allows to evaluate the anatomy and grade of stenosis of the arterial vessels and shows insensitivity to calcified plaques. First-pass cardiac MR perfusion imaging is also effective for the early diagnosis of CAD. Perfusion imaging allows monitoring blood circulation through the myocardium using a contrast agent. Therefore, it provides valuable information about the health of myocardial tissue [10].

Phase contrast (PC) sequences are special sequences that enable accurate evaluation of the blood flow at any location of the cardiovascular system, e.g., across the cardiac valves or cardiac shunts [11].

CMRI with magnetization tagging is useful to assess the mechanical function of individual portions of the heart [12], e.g., a quantitative evaluation of the intramyocardial contractile function.

The unique capability for tissue characterization is an important feature of CMRI. By means of late gadolinium enhancement (LGE) CMRI, it is possible to characterize myocardial scarring and inflammation. This is useful to assess the prognosis of myocardial infarction or nonischemic cardiomyopathies [11]. T1 and T2 mapping also provide reliable tissue characterization. T1 mapping is a robust and highly reproducible index that provides meaningful measurements reflecting important myocardial properties [13]. On the other hand, T2 mapping technique can accurately and reliably detect areas of myocardial edema. It is considered more beneficial than other modalities in patients with recent-onset heart failure and reduced left ventricular function [13].

## Challenges in cine CMRI

Dynamic CMRI is a technically challenging imaging modality. One of the main goals in this field of study is the improvement of efficiency in the acquisition procedure. Therefore, the challenge consists in accelerating the inherently slow data acquisition without compromising the high resolution and image quality requirements. As a direct consequence of its slowness, MRI traditionally shows significant limitations in imaging moving organs [1]. In fact, motion during the MRI scan process constitutes the major source of image degradation. Any movement, even in the case of small displacements, gives rise to characteristic artifacts in the reconstructed images due to the alteration in the k-space data. Among those undesired effects are image blurring, ghosting, and misregistration [14]. This aspect is particularly problematic in cine CMRI, where dealing with motion induced by heart beating and patient breathing remains one of the main challenges. Furthermore, other sources of motion should be considered, such as bulk motion resulting from voluntary or involuntary patient repositioning at the scanner. Thus, it can be assumed that the overall motion of the heart consists of three components: heart pumping, respiration, and any patient movement due to the lack of comfort during the scan.

### Respiratory motion

Breathing is the main source of motion and, therefore, of image degradation in CMRI [15]. The contraction and relaxation of the diaphragm and the intercostal muscles induce the heart to move rigidly throughout the respiratory cycle. The relationship between the heart motion and the superior-inferior displacement of the diaphragm is approximately linear, although there is a high intra- and inter-subject variability [14, 15]. For simplicity, respiration is usually considered a periodic process. However, it is well known that the respiratory-induced heart motion is different in inspiration and expiration due to lung hysteresis. Generally, the largest component of motion is in the inferior direction during inspiration [14]. As discussed below in the “Facing the challenges” section, a simple solution to deal with respiratory motion in CMRI consists in applying breath-holding (BH) acquisition protocols. It is important to note that these routines also affect the heart dynamics, leading to changes in the heart rate, which increases toward the end of the breath hold.

### Cardiac motion

As commented above, the motion induced by the own heart activity is another cause of image quality worsening in CMRI. The movement of the pumping chambers of the heart throughout the cardiac cycle is really complex [15]. More specifically, the left ventricle motion in the course of systole mainly comprises a longitudinal shortening, a radial contraction, and opposed rotations at the level of apex and base [14]. In current CMRI clinical protocols, k-space data are usually acquired along different cardiac cycles. For this reason, synchronization with the cardiac-induced motion is required. The electrocardiogram (ECG) signal is usually employed to this aim. It is common to assume that cardiac motion is periodic. However, this hypothesis is an excessive simplification, since many factors affect heart rate and motion differs between heartbeats. In this sense, it is worth mentioning that irregular cardiac rhythms (i.e., arrhythmias) hinder synchronized data acquisition and result in poor quality images or incomplete scans [1, 4, 6].

### Facing the challenges

Firstly, we consider the case of motion induced by the cardiac activity. Usually, the cardiac cycle is split into short frames to minimize the effect of the motion within each cardiac phase. ECG signal is commonly used for data synchronization purposes in a reliable way. This is known as ECG gating, and it can be carried out prospectively or retrospectively. In prospective cardiac gating, data are acquired over multiple cardiac cycles using the R wave from the ECG to trigger the acquisition. A set of k-space projections covering between 80 and 90% of the cardiac cycle is acquired repeatedly in each R-R interval, until enough k-space samples have been acquired [16]. This is done to deal with variations of the heart rate. The principal drawback of prospective ECG gating lies in the fact that a portion (10–20%) of the cardiac cycle is not included in the acquisition window. On the other hand, in retrospective cardiac gating, the k-space data are acquired in a continuous way and are timestamped to allow a posterior synchronization with the ECG signal. Regarding the ECG signal, it can be monitored and recorded during the scan or estimated from the acquired MR data. In this last case, the process is known as cardiac self-gating [17–19]. As stated above, image degradation may occur in patients with irregular cardiac rhythms due to the difficulty of achieving a proper cardiac gating [6]. For this reason, some reconstruction methods include protocols to deal with arrhythmias. As an example, Chitiboi et al. simultaneously reconstruct different arrhythmic cycles in a five-dimensional image space [20], in which a classification of irregular cardiac cycles constitutes an extra dimension. However, the simplest solution consists in discarding atypical cardiac cycles [21], a practice that worsens the efficiency of the MRI protocol because of the rejected data.

Simple solutions to deal with respiratory motion are either BH procedures or navigator-based acquisitions. Breath holding requires patient cooperation to replicate the same position between successive BH to avoid misalignment and artifacts in the images. Even if the BH reproducibility is adequate, the diaphragm can drift considerably at the end of long apneas. Improvements in MRI technology and acquisition sequences have enabled to complete the CMRI study in a single BH, although SNR and spatiotemporal resolution of the images may be compromised. For this reason, the acquisition is commonly performed along multiple BH. In addition, BH procedures are severely hindered by non-cooperating patients, either children or pathological patients with apnea difficulties. The alternative is to use respiration monitoring by means of a chest belt with pressure sensors, or the acquisition of navigator pulses as in [22]. Both BH and navigator-based procedures compromise the scan efficiency. Thus, free-breathing (FB) acquisition procedures, with retrospective respiratory gating and motion estimation and compensation (ME-MC) approaches, are of great interest. As in the case of cardiac gating, respiratory motion can be extracted from the acquired MR data, i.e., respiratory self-gating [21, 23–25].

Another option to avoid the problems of traditional breath-holding approaches is real-time cine CMRI. However, common real-time sequences lead to a worsening of the quality of the images. Normally, the spatial and temporal resolution is compromised, and signal-to-noise ratio (SNR) is lower, since acquisition must be carried out during time intervals of 100 ms or less to avoid intra-scan motion. Because of its great clinical benefit, many reported studies have tried to overcome these drawbacks in FB real-time techniques.

#### Cardiac and respiratory self-gating approaches

Among the proposed cardiac self-gating approaches, Crowe et al. present a self-gated rectilinear TrueFISP cine sequence [18]. The retrospectively gated TrueFISP sequence is modified to acquire a short second echo after the readout and phase gradients are rewound. The gating signal is then derived from this second echo. Kramer et al. combine golden-ratio radial acquisition with retrospective cardiac gating provided by a 1D navigator acquired at fixed intervals [19]. Meanwhile, Larson et al. propose three strategies to extract the cardiac signal directly from the MR data using radial sampling: echo peak magnitude, kymogram, and 2D correlation [17].

Larson et al. also propose a respiratory self-gating strategy [26] based on radial sampling. In this case, the interleaved radial k-space sampling provides low-resolution images in real time during the FB acquisition. These images are compared to target expiration images, and only the raw data producing images with high correlation to the target images are included in the final high-resolution reconstruction. Uribe et al. derive the breathing motion using a center k-space profile, which is repeatedly acquired, and adjust the acquisition scheme to reacquire motion-corrupted data [23]. Peters et al. [22] propose the use of two navigators (NAVs), one placed prior to the QRS and another 500 ms after the QRS complex, after systole. In [24], Piccini et al. propose a respiratory self-gating method based on 3D spiral phyllotaxis sampling with superior-inferior (SI) projections acquired at the beginning of each interleave. The blood pool is detected from the 1D-FFT of these SI projections by means of a segmentation procedure, and its motion is computed using cross-correlation. A different approach is suggested in [25, 27], where Usman et al. introduce manifold learning to estimate the respiratory signal directly from undersampled radial MR data.

In addition to the abovementioned approaches, there are also proposals that estimate both cardiac and respiratory signals. In [28], Liu et al. use multiecho hybrid radial sampling with Cartesian mapping of the k-space center along the slice encoding direction. This sampling scheme provides intensity-weighted position information, from which both respiratory and cardiac motions are derived. Pang et al. [21] propose simultaneous cardiac and respiratory self-gating through SI readouts inserted at regular intervals during acquisition. The signals are estimated by means of the PCA of the 1D-FFT of the SI projections.

## Speeding up CMRI

### Fast acquisition

Considerable efforts are carried out to make CMRI faster. As commented above, the objective is to achieve a high imaging speed while a good image quality is preserved. In this sense, ultrafast imaging refers to efficient scan techniques that use a high percentage of the scan time for data acquisition [29]. The improvement of patient comfort is the most important benefit of fast acquisitions. Moreover, motion effects during a shorter scan are minimized. Therefore, it may be possible to make the scan sessions more effective and comprehensive.

**Fig. 2:**
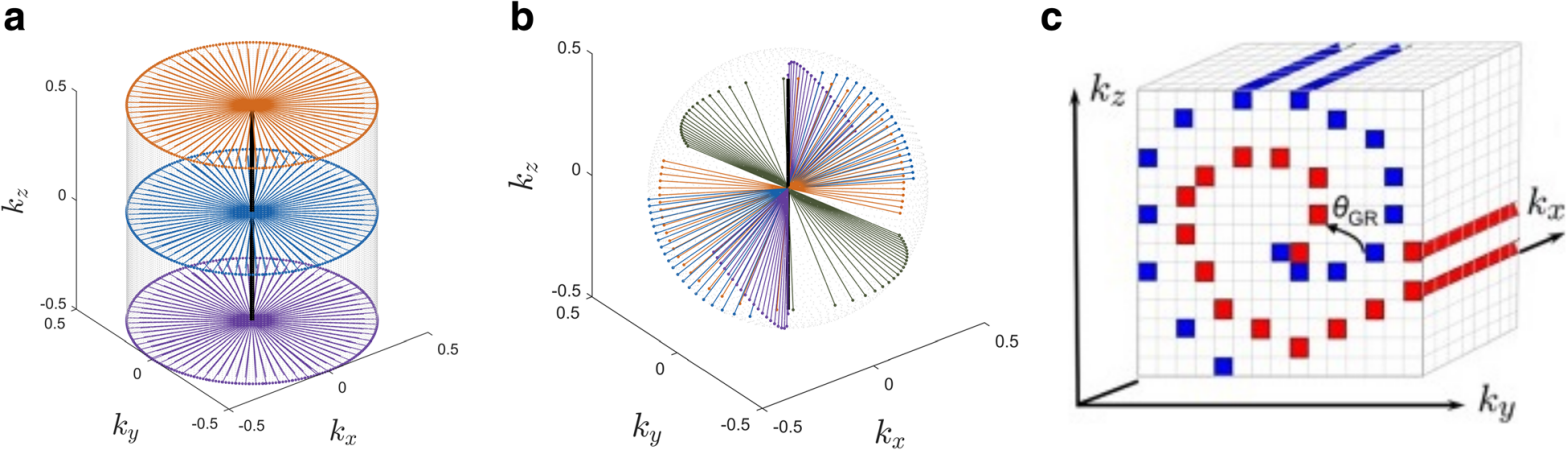
Examples of radial 3D k-space sampling schemes. **a** Stack-of-stars (SoS). **b** Spiral phyllotaxis. **c** Golden angle Cartesian acquisition with Spiral Profile ordering (G-CASPR) [38]

Parallel imaging (PI) can be used to improve acquisition times [30, 31]. The information about coil sensitivities can be incorporated to enhance the results. Furthermore, the use of efficient k-space sampling strategies has been widely investigated to reduce acquisition time and generate high SNR images. Useful trajectories are echo planar imaging (EPI) [32] and a variety of non-Cartesian sampling patterns, such as golden-angle radial schemes [33], stack-of-stars (SoS) [34], or spirals phyllotaxis [35]. These non-Cartesian trajectories with denser sampling at the center of k-space have shown certain advantages for self-gating approaches, as well as robustness against motion artifacts. However, a gridding procedure is required to interpolate non-Cartesian data onto a rectangular grid for the posterior application of the FFT in the reconstruction process. This step increments substantially the reconstruction times [36]. To overcome this drawback, different pseudo-radial trajectories have been recently proposed, such as VDRad [37], G-CASPR [38], CASPR-Tiger [39], and ROCK [40], among others. These trajectories acquire data along radial-like projections on a Cartesian grid and have the advantage of low computational complexity [39]. Figure 2 shows some of the aforementioned 3D radial sampling schemes.

Compressed sensing (CS) [41] is also applied to MRI in order to speed up the acquisition procedure [42]. These accelerated methods are based on the incoherent subsampling of the k-space data. Then, the reconstruction procedure is formulated by means of an unconstrained nonlinear optimization problem. As for the sampling patterns, CS procedures have shown better results for trajectories with more density of samples in the central region of k-space [43].

Low-rank procedures are an alternative to CS. Low-rank matrix completion extends the idea of CS to matrices, enabling recovery of missing or corrupted entries under low-rank and incoherence conditions [44]. Thus, sparse images can be represented by low-rank matrices and undersampling becomes possible.

### Fast reconstruction

Not only the reduction of the scan times is important, but also the shortening of reconstruction times [45]. Specifically, fast reconstruction is essential in real-time CMRI. Coil array compression is crucial to reduce the computational cost of reconstructions [46–48]. Dedicated computing devices, graphics processing units (GPUs) in particular, provide significant efficiency boosts and, therefore, improve the reconstruction speed [49, 50]. Moreover, there are several frameworks and libraries with efficient and specialized reconstruction packages, such as the Gadgetron [51], the Berkeley advanced reconstruction toolbox (BART) [52], and the recently proposed OpenCLIPER [53]. Another discipline to consider in this field due to its high potential and generalization capabilities is machine learning, deep learning (DL) more specifically. Some recent studies have shown promising achievements using DL approaches [54–58]. More details about DL-based reconstruction methods are included in the “Deep learning and beyond” section.

## Classification and reconstruction techniques

### Multi-slice 2D cine CMRI

A review of the most relevant reconstruction techniques proposed in 2D cine CMRI is included below.

#### Multiple-BH methods

Classical approaches in cine CMRI reconstruction attempt to increase data acquisition speed by reducing the amount of acquired data. In [59], two model-based methods for accelerated dynamic CMRI reconstruction are proposed, namely, k-t BLAST (broad-use linear acquisition speed-up technique) and k-t SENSE (SENSitivity Encoding) for single or multiple receiver coils, respectively. Data correlations in both k-space and time domains are exploited to recover unacquired data. In the same line, the idea of GRAPPA combined with sliding window techniques is applied in k-t GRAPPA [60] to interpolate the missing data in k-t space, in this case, without requirement for acquisition of training data and calculation of sensitivity maps.

Lustig et al. [61] suggest k-t SPARSE, a CS-based method exploiting both spatial and temporal sparsity of the dynamic CMRI sequences, which leads to a 7-fold frame-rate acceleration. Specifically, the wavelet and Fourier transforms are used in the spatial and temporal dimension, respectively. Among CS-based techniques, k-t FOCUSS [62, 91] proposes a ME-MC approach based on the use of a high-quality reference frame and a block matching algorithm applied independently to each frame. Temporal discrete Fourier transform is used to achieve sparse representation of the temporal variations in cardiac images. In contrast, the motion-adaptive spatiotemporal regularization method (MASTeR) [67] does not require a reference frame. Spatial sparsity is modeled by means of wavelet transform, whereas motion-adaptive transforms are used to model the temporal sparsity in images. Motion between adjacent frames is estimated in forward and backward directions from an initial reconstruction.

An alternative to these pairwise approaches for ME is presented in [78], where Royuela-del-Val et al. propose a more robust group-wise (GW) approach. Specifically, a non-rigid GW registration method based on a B-spline deformation model is suggested. Thus, the whole sequence is registered at once to compensate for the naturally induced motion of the heart. Departing from an initial reconstruction, the groupwise CS (GW-CS) method obtains refined reconstructed images and estimated motion information in an iterative way. This methodology was subsequently refined by introducing a new sparse regularization term, the Jacobian weighted temporal total variation (JW-tTV) [82].

A different CS-based reconstruction method is presented in [73]. In this proposal, Wang et al. incorporate a dictionary learning (DicL) approach. Mohsin et al. [83] suggest a patch smoothness regularization procedure (PRICE) for implicit inter-frame MC without requiring reference frames or complex motion models.

In contrast to the schemes that rely on the sparsity in Fourier space, Lingala et al. propose the k-t SLR method [65], in which a compact representation of the data in the Karhunen-Louve transform (KLT) domain is used to exploit the correlations in the dataset. The problem is posed as a spectrally regularized matrix recovery problem.

In the group of low-rank procedures, k-t PCA method is presented in [63], where Pedersen et al. suggest a generalization of k-t BLAST/SENSE by constraining the reconstruction using principal component analysis (PCA). Christodoulou et al. [64] propose the use of anatomical constraints to improve SNR and to reduce artifacts in partially separable function (PSF) reconstructions. In [76], the model consistency condition (MOCCO) technique is introduced. Low-rank temporal signal models are pre-estimated from training data and used in the reconstruction procedure.

Other proposals are based on a combination of low-rank matrix completion and CS theories. In these methods, the authors divide dynamic imaging in a low-rank (L) component and a sparse (S) component (L+S decomposition), also referred to as robust principal component analysis (RPCA). The reconstruction is formulated as an optimization problem minimizing a cost function with a data fidelity term and different regularization terms. Otazo et al. [44] formulate a multicoil L+S reconstruction, where the L component models the temporally correlated background and the S component models the organ motion. The nuclear norm and l1 norm are used as the convex surrogate functions for the rank function and l0 norm, respectively, in the optimization problem. In [72], k-t RPCA method is proposed, which uses the Fourier transform as the sparsifying transform in the temporal direction and the alternating direction methods of multipliers (ADMM) framework to solve the minimization problem. Another proposal is [84], in which the convex optimization problem is solved by a scalable and fast algorithm based on the inexact augmented Lagrange multipliers (IALM). In [85], Xu et al. introduce an alternating direction method (NADM) for nonconvex RPCA low-rank matrix approximation. Roohi et al. [86] formulate a higher dimensional L+S tensor reconstruction problem and also use ADMM to solve the optimization problem. More recently, Tolouee et al. [89] proposed an L+S decomposition coupled with a registration algorithm for ME using a reference dataset free of respiratory motion. This reference is derived from the measurements themselves.

#### Single-BH methods

A CS-based method is presented in [74] to acquire four short axis (SA) and three long axis (LA) views of the heart in a single BH. A Cartesian acquisition pattern is used, which limits the spatiotemporal resolution and produces aliasing problems along the phase encoding direction. The temporal resolution determines the acquisition and must be set before the scan.

Royuela-del Val et al. [75] proposed the kt-WiSE method based on GW-CS with golden radial acquisition pattern. In a posterior study [87], the authors adapt their previously proposed JW-tTV methodology to golden radial k-space trajectories for application to whole-heart Single-BH cine CMRI.

In [79], a locally low-rank (LLR) framework is combined with temporal finite difference (FD) and PI. Golden-angle radial sampling is used for acquisition of multiple 2D slices in a single BH. However, the reconstructions show spatiotemporal blurring. Authors attribute this effect to the eddy current-induced image artifacts.

#### FB methods

In [69], a generalized motion correction formulation is directly incorporated into the CS reconstruction for 2D respiratory self-gated FB cine CMRI. Acquired FB golden radial k-space profiles are binned into different motion states, such that respiratory motion within each predefined state is not significant so as to produce artifacts in the reconstructed images. Separate motion compensated CS (MC-CS) reconstructions are performed for every motion state. An extended version of this method was presented in [92], in which Usman et al. combine the previously proposed MC-CS framework with parallel imaging to achieve further acceleration. In another contribution from the same research group [93], they introduce a manifold learning method to estimate both cardiac and respiratory navigator signals from the acquired data itself, allowing retrospective self-gated cine reconstruction.

The XD-GRASP framework [80] has also been applied to 2D FB cine CMRI. It is based on the continuous acquisition of k-space data following a golden-angle sampling pattern. Instead of applying some kind of MC, dynamic data is retrospectively sorted into extra cardio-respiratory motion states. The resulting multidimensional dataset is reconstructed by means of a CS approach, in which sparsity along both cardiac and respiratory dimensions is simultaneously enforced.

Among real-time cine CMRI techniques, in [94], a denoising algorithm for SNR enhancement is proposed. Hansen et al. [66] suggest a general reconstruction framework of cine CMRI from a real-time acquisition, with data acquired over multiple cardiac cycles during FB. The proposed reconstruction method is based on a temporal multi-resolution scheme and combines PI with a MC strategy based on non-rigid registration. In a posterior study [70], the same authors attempt to further shorten the required acquisition time by employing a non-linear reconstruction step. Feng et al. propose the application of k-t SPARSE-SENSE method [68], based on a combination of k-t SPARSE and sensitivity encoding, to real-time CMRI. Meanwhile, Schmidt et al. [71] suggest a CS-based reconstruction with k-t regularization for highly accelerated real-time cine CMRI as a potential alternative providing high spatiotemporal resolution. Poddar et al. [77] introduce a real-time acquisition and reconstruction method termed SToRM (SmooThness Regularization on Manifolds). In this case, image frames are modeled as points on a smooth and low-dimensional non-linear manifold. The entire dynamic dataset is recovered by means of a manifold smoothness regularized reconstruction problem. Chen et al. present a parallel scheme for online reconstruction in [81], where the first frame is used to guide all the subsequent reconstructions to exploit the temporal redundancy. Dynamic total variation (dTV) is introduced to exploit the sparsity in both spatial and temporal domains. An accelerated reweighted least squares algorithm is used to solve the reconstruction. In [88], Wang et al. propose the combination of parallel DicL and dTV (PDLDTV) for real-time dynamic CMRI reconstruction and use a primal-dual algorithm to achieve the required high reconstruction speed. Recently, a radial acquisition with k-space variant reduced-FOV reconstruction is suggested by Li et al. in [90]. A correlation imaging framework is introduced to convert PI reconstruction into the estimation of correlation functions. Cartesian data is directly calculated from the linear combination of its neighboring radial samples in a k-space variant fashion.

Table 1 shows an overview of the above mentioned methodologies to close the "Multi-slice 2D cine CMRI" section.

**Table 1:** Summary of reconstruction techniques for 2D cine CMRI

Authors	Year	Mode	Method	Salient features	Performance
Tsao et al. [59]	2003	Multi-BH	k-t BLAST, k-t SENSE	Model-based method exploiting data correlations to recover unacquired samples. Cartesian sampling	4-fold acceleration. Spatial res., 2.42 × 2.52 mm^2^ (slice thickness 10 mm). Temporal res., 26 ms
Huang et al. [60]	2005	Multi-BH	k-t GRAPPA	GRAPPA combined with sliding window techniques for missing data interpolation. Cartesian sampling	AF = 7. Reduction factor, 5.17. Spatial res., 1.77 × 1.82 mm^2^ (slice thickness, 6 mm). Number of phases, 14. Reconstruction time, 4 s per frame
Lustig et al. [61]	2006	Multi-BH	k-t SPARSE	CS-based method exploiting spatial and temporal sparsity of data. Cartesian sampling	7-fold frame-rate acceleration. Spatial res., 2.5 × 2.5 mm^2^ (slice thickness, 9 mm). Temporal res., 40 ms. Reconstruction time, 1 h per 64 × 64 × 64 scene
Jung et al. [62]	2009	Multi-BH	k-t FOCUSS	CS method with ME-MC based on block matching. Cartesian sampling	AF = 6. Spatial res., 1.25 × 1.17 mm2 (slice thickness, 5 mm). 25 cardiac phases
Pedersen et al. [63]	2009	Multi-BH	k-t PCA	Generalization of k-t BLAST/SENSE using PCA temporal constraint. Cartesian sampling	Myocardial perfusion images acquired in a pig. 8-fold acceleration. Spatial res., 1.25 × 1.25 mm^2^ (slice thickness, 10 mm). 64 frames
Christodoulou et al. [64]	2010	Multi-BH	PSF	Partially separable function reconstruction with anatomical constraints	Data of rat hearts. Spatial res., 390 μm in-plane (slice thickness, 1.5 mm). Temporal res., 15 ms
Lingala et al. [65]	2011	Multi-BH	k-t SLR	Low-rank structure using KLT to exploit the sparsity. Cartesian sampling	Cardiac perfusion MRI data. AF = 11. Matrix size, 90 × 190
Hansen et al. [66]	2012	FB real-time	–	Temporal multi-resolution scheme combining PI with MC based on nonrigid registration. Cartesian and golden-angle radial sampling	2-fold PI acceleration. Spatial res., 1.4–1.5 × 1.9–2 mm^2^ (Cartesian), 1.4–1.5 × 1.4–1.5 mm^2^ (golden angle radial), slice thickness, 6 mm. Temporal res., 30 ms
Asif et al. [67]	2013	Multi-BH	MASTER	CS with ME-MC based on motion-adaptive spatio-temporal regularization. Cartesian sampling	Retrospective downsampling with reduction factor up to 10. Spatial res., 1.56 × 1.37 mm^2^ (slice thickness, 12 mm). 16 cardiac phases
Feng et al. [68]	2013	FB real-time	k-t SPARSE SENSE	Combination of CS and PI for real-time imaging. Cartesian sampling	8-fold acceleration. Spatial res., 2.3 × 2.3 mm^2^ (slice thickness, 8 mm). Temporal res., 43.2 ms. Offline reconstruction time, 4.6 min per slice
Usman et al. [69]	2013	FB	MC-CS	Generalized MC in CS reconstruction. Respiratory motion self-gating by low resolution virtual 2D navigator images. Golden angle radial sampling	AF = 4–6. Spatial res., 1.5–2 × 1.5–2 mm^2^. 20 cardiac phases. Temporal res., 30–40 ms. Reconstruction time, 2–2.5 h.
Xue et al. [70]	2013	FB real-time	–	SPIRiT non-linear reconstruction with spatial-temporal regularization (Harr wavelet transformation) and ME-MC based on non-rigid registration. Cartesian time-interleaved sampling	Scan time, 16–20 s per acquired slice. PI reduction factor of *R* = 4. Spatial res., 1.3–1.8 × 1.8–2.1 mm^2^ (slice thickness, 8 mm). 30 cardiac phases. Temporal res., 34.3 ± 9.1 ms. Inline reconstruction time (Gadgetron), 80–120 s per slice
Schmidt et al. [71]	2013	FB real-time	rtCS11	Real-time CS-based reconstruction with k-t regularization. Cartesian sampling	Scan time, 1 heartbeat. AF = 10.9. Spatial res., 1.7 × 1.7 mm^2^ (slice thickness, 6 mm). Temporal res., 30 ms. Online reconstruction
Trémoulhéac et al. [72]	2014	Multi-BH	k-t RPCA	L+S decomposition based on RPCA with temporal FT. Variable density Cartesian and pseudo-radial sampling	AF = 8. Matrix size, 128 × 128 (90 frames). Reconstruction time, 10 min
Wang et al. [73]	2014	Multi-BH	–	CS-based reconstruction with DL. Retrospective Cartesian undersampling	AF up to 8. Matrix size, 150–256 × 256–304 (14–26 frames). Reconstruction time, 11.3–24.3 min
Vincenti et al. [74]	2014	Single-BH	–	CS-based method with Cartesian acquisition	AF = 11. 3 long-axis and 4 short-axis views. Spatial res., 1.5 × 1.5 mm^2^ (slice thickness, 6 mm). 24 cardiac phases. Temporal res., 30 ms. BH duration, 14 s
Royuela-del Val et al. [75]	2015	Single-BH	kt-WiSE	MC-CS based on GW registration with SENSE. Golden angle radial sampling	AF = 16. Spatial res., 2 × 2 mm^2^, (slice thickness, 8 mm, 12 slices). 16 cardiac phases Temporal res., 46.4 ms. BH duration, 11.1 s
Velikina et al. [76]	2015	Multi-BH	MOCCO	Pre-estimated low-rank temporal signal models. Variable density Cartesian sampling	AF up to 15. Spatial res., 1 × 1.7 mm^2^ (26 and 30 cardiac phases)
Otazo et al. [44]	2015	Multi-BH	–	L+S reconstruction. Cartesian sampling for cardiac cine. Radial sampling for abdominal and breast DCE-MRI	8-fold acceleration. Spatial res., 1.25 × 1.25 mm^2^ (slice thickness, 8 mm). 24 temporal frames
Poddar and Jacob [77]	2016	FB real-time	SToRM	Manifold smoothness regularized reconstruction with radial sampling	Scan time, 42 s per slice. Spatial res., 1.17 × 1.17 mm^2^, (slice thickness, 5 mm, 5 slices). Temporal res., 42 ms. Reconstruction time, 24 min (l2-SToRM) and 4.9 h (l1-SToRM)
Royuela-del Val et al. [78]	2016	Multi-BH	GW-CS	CS method with ME-MC based on non-rigid GW registration and Cartesian sampling	AF up to 12. Spatial res., 2 × 2 mm^2^ (slice thickness, 8 mm). 16 cardiac phases
Miao et al. [79]	2016	Single-BH	LLR + FD	Locally low rank with temporal finite difference and PI using golden-angle radial sampling	AF = 19–23. Spatial res., 2 × 2 mm^2^, (slice thickness, 8 mm, 12 SA slices). Temporal res., 40 ms (19–20 time frames). BH duration, 9–13 s
Feng et al. [80]	2016	FB	XD-GRASP	CS-based reconstruction of extra cardio-respiratory motion states. Continuous acquisition with golden-angle trajectory	Scan time, 20 s per slice. AF = 16. Spatial res., 2 × 2 mm^2^, (slice thickness, 8 mm, 3 SA + 1 4CH slices). Temporal res., 45 ms. 18–26 cardiac phases and 10–16 respiratory phases
Chen et al. [81]	2016	FB real-time	–	Parallel online reconstruction using dTV and accelerated reweighted least squares algorithm. Radial sampling	Matrix size, 256 × 256 × 24. Reconstruction time, 33.1 s
Royuela-del Val et al. [82]	2017	Multi-BH	JW-tTV	CS-MC method using Jacobian weighted temporal TV as sparse regularization term. Cartesian sampling	AF = 12. FOV = 320 × 320 mm^2^, (slice thickness, 8 mm). 30 cardiac phases
Mohsin et al. [83]	2017	Multi-BH	PRICE	Implicit inter-frame MC based on patch smoothness regularization. Cartesian sampling	Scan time, two heartbeats per slice. AF = 6. Spatial res., 2.5 × 2.5 mm^2^. 1 slice, 20 temporal frames (16 lines per frame). Reconstruction time, 7 min
Chen et al. [84]	2017	Multi-BH	–	L+S method. RPCA inverse problem solved by IALM. Cartesian and pseudo-radial sampling	AF = 6. Spatial res., 1.25 × 1.25 mm^2^, (slice thickness, 10 mm). 1 slice, 30 temporal frames. Reconstruction time, 2–2.2 min
Xu et al. [85]	2017	Multi-BH	G-NADM, L-NADM	L+S method with NADM for nonconvex RPCA	Matrix size, 256 × 256. 1 slice, 24 temporal frames. Reconstruction time, 3–3.3 min
Roohi et al. [86]	2017	Multi-BH	k-t MLSD	Multi-dimensional L+S decomposition method. Cartesian and radial sampling	Sampling rate, 0.25. Spatial res., 1.35 × 1.05 mm^2^, (slice thickness, 10 mm). 25 temporal frames (66 bpm). Reconstruction time, 26.64 s per slice
Royuela-del Val et al. [87]	2017	Single-BH	JW-tTV-GR	Adaptation of JW-tTV to golden radial acquisition pattern. Whole-heart coverage	AF = 16. 12–14 SA slices. Spatial res., 2 × 2 mm^2^ (slice thickness, 8 mm). 13–16 cardiac phases. Temporal res., 46.4 ms. BH duration, 10–13 s
Wang et al. [88]	2017	FB real-time	PDLDTV	Parallel DicL and dTV method using a primal-dual algorithm. Radial sampling	Sampling rate, 70% 1st frame, 15% rest. Matrix size, 256 × 256, 24 temporal frames. Reconstruction time, 2 min
Tolouee et al. [89]	2018	Multi-BH	–	L+S method with MC based on a deformable registration method. Cartesian sampling	AF = 12. Spatial res., 1.35 × 1.05 mm^2^, (slice thickness, 10 mm). Temporal res., 25 ms
Li et al. [90]	2018	FB real-time	–	k-space variant reduced-FOV reconstruction. Radial sampling	Spatial res., 1.7 mm^2^, (slice thickness, 8 mm). Temporal res., 40 ms. Reconstruction time, 2 s per frame

### 3D cine CMRI

Next, a survey of reconstruction techniques for 3D cine MRI is presented.

#### Single-BH methods

Wech et al. [95] propose a CS-based reconstruction method using a 3D SoS undersampled trajectory for dynamic MRI of the whole heart in a single BH of 27 s, with non-isotropic spatial resolution (2.1 × 2.1 × 8 mm^3^) and temporal resolution of 40.5 ms. The authors conclude that an acceleration factor (AF) of 10.7 with respect to a fully sampled radial SoS acquisition (6.8 with respect to a Cartesian 3D acquisition on the according grid) could be achieved without compromising the diagnostic relevance.

In [97], Jeong et al. perform a validation study of 3D cine MRI of the heart in a single BH using kat-ARC, which is an auto-calibrating PI method for Cartesian sampling. It uses a motion-adaptive k-t synthesis kernel that exploits spatial and temporal correlations and selects a temporal window to reduce motion artifacts. The reported results, with 2 × 2 × 5 mm^3^ spatial resolution and mean required apnea of 22 s, do not show clinically significant differences with standard 2D cine CMRI.

Recently, Wetzl et al. [99] present a 3D Single-BH approach with a nearly isotropic resolution of 1.9 × 1.9 × 2.5 mm^3^, temporal resolution 42–48 ms, and a BH duration of 19 s for an acquisition covered just the left ventricle and 32 s for the whole heart. A Cartesian sampling pattern based on the spiral phyllotaxis and a CS reconstruction method are used to achieve high AFs.

#### FB methods

In 2010, Liu et al. introduce a FB 3D cine CMRI method with both respiratory and cardiac self-gating based on a SoS acquisition strategy [28]. Cardiac and respiratory motions are estimated from the acquired data itself. The estimated signals are used in a retrospective double-gating scheme, in which only 50% of data is used for the subsequent reconstruction. The same authors, in a posterior study [101], explore an alternative respiratory self-gating signal called the *Z* intensity-weighted position (ZIP).

In [39], Usman et al. propose a self-gated Cartesian approach for 3D cine CMRI with isotropic resolution and no data rejection. Data is acquired continuously under FB using CASPR-Tiger trajectory, CArtesian acquisition with Spiral PRofile ordering and Tiny golden-angle step for eddy current reduction. 4D volumes (3D + cardiac phase) are reconstructed using a soft gating technique and iterative SENSE with tTV. Han et al. also propose a self-gated Cartesian methodology in [40]. Although it is originally conceived for application to abdominal MRI, its applicability to cine CMRI would be almost straightforward. It is based on a 3D rotating Cartesian k-space (ROCK) reordering method. This acquisition scheme allows for respiratory motion estimation and retrospective data binning in multiple respiratory states. The reconstruction is formulated as a CS-based method with spatial and temporal regularization and PI.

Another free-running (i.e., self-navigated and FB) approach for 4D CMRI reconstruction is proposed in [96]. The data acquisition scheme is based on the 3D spiral phyllotaxis trajectory and incorporates SI projections for respiratory self-navigation. This technique provides high isotropic spatial resolution allowing both functional imaging of the heart and coronary MRA, in which contrast agent injection is not a requirement.

Recently, the XD-GRASP method has been extended to reconstruct 5D cardiac and respiratory motion-resolved whole-heart cine MRI [100]. In this case, the data acquisition scheme and respiratory motion extraction previously proposed by Coppo et at. [96] are adopted. The 5D domain refers to the three spatial variables plus cardiac phase and respiratory phase. In [98], Menchón-Lara et al. introduce a 3D GW cardio-respiratory ME-MC technique in an analogous reconstruction framework. Moreover, the authors incorporate an efficient multi-resolution scheme, which leads to significant improvements in the quality of the recovered image series.

Table 2 summarizes the reconstruction techniques for 3D cine CMRI.

**Table 2:** Summary of reconstruction techniques for 3D cine CMRI

Authors	Year	Mode	Method	Salient features	Performance
Liu et al. [28]	2010	FB	–	Respiratory and cardiac self-gating. SoS acquisition. Temporal filtering is applied along cardiac phases. Non-isotropic reconstructions with data rejection	10–14 SA and 8 2CH–4CH slices. Spatial res., 1.25–1.33 mm^2^, slice thickness, 10 mm (SA), 8 mm (2CH and 4CH). Temp. res., 44 ms (SA), 35 ms (2CH and 4CH)
Wech et al. [95]	2014	Single-BH	–	CS-based method using undersampled SoS acquisition. Non-isotropic spatial resolution	AF = 10.7. Spatial res., 2.1 × 2.1 × 8 mm^3^ (12 slices). Temp. res., 40.5 ms. BH duration, 27 s
Coppo et al. [96]	2015	FB	–	Free-running method based on 3D spiral phyllotaxis sampling. Respiratory self-gating and retrospective binning	AF = 9.8. Scan time, 14.28 min. Spatial res., 1.15 mm^3^. Temp. res., 20 ms (43 frames). Reconstruction time, 6 h
Jeong et al. [97]	2015	Single-BH	kat-ARC	Auto-calibrating PI method for Cartesian sampling	AF = 8. Spatial res., 2 × 2 × 5 mm^3^. Temp. res., 36–70 ms. BH duration, 22 s
Usman et al. [39]	2017	FB	CASPR-Tiger	Free-running CS method using iterative SENSE with tTV. Self-gated Cartesian acquisition with spiral profile ordering and tiny golden angle step. No data rejection	AF = 3.5–4. Scan time, 4–5 min. Spatial res., 2 mm^3^ (isotropic). Temporal res., 31–70 ms (16 cardiac phases). Reconstruction time, 2.5 h
Han et al. [40]	2017	FB	ROCK	Self-gated CS method with spatial and temporal regularization and PI using a Cartesian k-space reordering method	Abdominal MRI. Scan time, 5 min. Spatial res., 1.2 × 1.2 × 1.6 mm^3^. 8 respiratory phases. Reconstruction time (BART), 10 min
Menchón et al. [98]	2017	FB	MC-XD	CS method with cardio-respiratory ME-MC based on 3D nonrigid GW registration. Efficient spatial multiresolution strategy. Retrospective 3D spiral phyllotaxis sampling	AF = 24.38–34.8. Spatial res., 1 mm^3^ (isotropic). Temp. res., 43–50 ms (20 cardiac phases and 4 respiratory phases). Reconstruction time, 1.42 h
Wetzl et al. [99]	2018	Single-BH	–	CS method with non-linear, iterative SENSE using Cartesian sampling pattern based on the spiral phyllotaxis. Nearly isotropic spatial resolution	AF = 23. Spatial res., 1.6 × 1.9 × 2.3 mm^3^. Temp. res., 42–48 ms. BH duration, 32 s. Reconstruction time, 10 min
Feng et al. [100]	2018	FB	5D-GRASP	Extension of the XD-GRASP method for 3D spiral phyllotaxis trajectory with respiratory self-gating	AF = 18.3. Scan time, 14.28 min. Spatial res., 1.15 mm^3^ (isotropic). Temp. res., 40–50 ms (20 cardiac phases and 4 respiratory phases). Reconstruction time, 6.8 h

### Brief discussion

Nowadays, multi-slice 2D cine CMRI has become the standard imaging modality for functional studies of the heart in clinical practice. In standard cine scans, multiple 2D slices covering the volume of the heart are obtained. However, multi-slice 2D cine approaches usually have anisotropic spatial resolution, typically with low through-plane (slice thickness) resolution. Furthermore, data can only be acquired in a specific geometry, such as short-axis (SA), two-chamber (2CH), or four-chamber (4CH) views, which requires a planning stage before starting the scan (view Fig. 1). This fact does not allow for retrospective reformatting to arbitrary orientations. Moreover, 2D cine CMRI may be adversely affected by misalignment between slices acquired during different apneas with the usual multi-BH acquisition procedures. Although 2D Single-BH procedures have been explored, the achieved SNR or resolution within a comfortable BH period could be considered insufficient for many applications. In any case, BH procedures are inadequate for patients with respiratory distress syndrome or with other difficulties for respiratory suspension and for non-collaborative patients. Therefore, FB methodologies are preferable and more suitable in most of the cases.

3D cine CMRI avoids some of the aforementioned drawbacks of the multi-slice 2D cine modality. It provides increased SNR and large spatial coverage. Thanks to the isotropic spatial resolution, reconstructed volumes can be reformatted into any desired orientation. Thus, there is no need to perform a previous planning stage, and the overall scan time is reduced. However, 3D acquisitions also require robust strategies to mitigate the effect of motion in the reconstructed images. In addition, the excitation of a 3D volume also affects the contrast between myocardium and the blood pool, given the diminished portion of unsaturated blood entering the imaging volume. Some of the 3D approaches [28, 39] point out this issue and suggest contrast agent injection to improve the contrast. However, other studies [96, 100] maintain that contrast agent injection is not required.

**Fig. 3:**
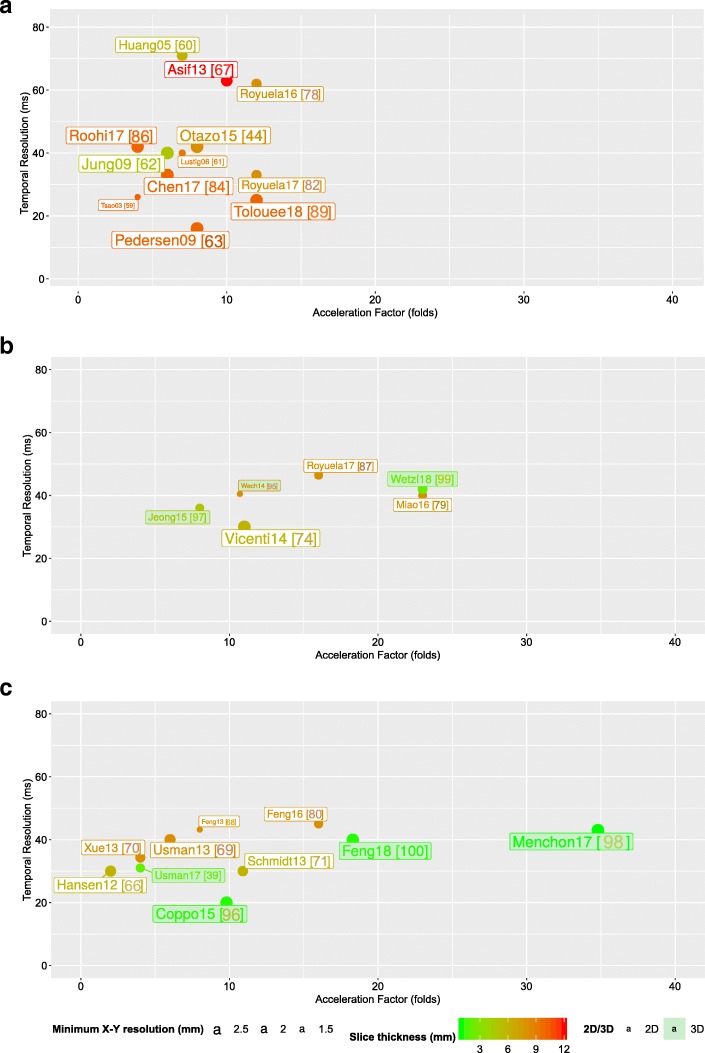
Graphical representation of performance. **a** Multi-BH reconstruction techniques. **b** Single-BH reconstruction techniques. **c** FB reconstruction techniques. Temporal resolution (ms) versus AF. Text boxes indicate first author, publication year, and reference in brackets. Shaded text boxes refer to 3D approaches. (Lowest) in-plane spatial resolution (mm) is codified varying the size of font and markers. Slice thickness (mm) is depicted using a color code

As for the different techniques, it is not easy to establish a comparative analysis. Tables 1 and 2 include the performance of each proposal in terms of AF, spatiotemporal resolution of images, and reconstruction times. Additionally, Fig. 3 shows a graphical representation of the performance of different methods for comparison purposes. Specifically, temporal resolution (ms) vs. AF is depicted for multi-BH, Single-BH, and FB reconstruction approaches separately. In some cases, when AF is not reported in the corresponding publication, it has been estimated from available data. In a similar way, the temporal resolution of the dynamic sequences reconstructed has been approximated assuming an average heart rate of 60 bpm when it is not reported. Shaded boxes are used to differentiate between 2D and 3D techniques. Moreover, graphics include information about the in-plane spatial resolution (mm) and slice thickness (mm) by using different sizes and colors. In general, CS and low-rank algorithms show potential for further acceleration of the acquisitions. However, these procedures involve longer reconstruction times. Thus, GPUs and specialized frameworks play an important role for reducing the reconstruction times.

## Deep learning and beyond

Reconstruction of cine CMRI will remain an active area of technological development. There is still room for improvement in motion detection and modeling, which would result in significant enhancement of image quality. In particular, dealing with irregular motion patterns will be a key aspect. Further progress in the reduction of scan and reconstruction times is also required for future works. Moreover, there is a great interest to transition to MRI guidance for cardiac interventions [45]. To this end, evolution of real-time imaging is crucial. It is worth noting that the role of machine learning (deep learning, in particular) is also promising for reconstruction of cardiac cine MRI.

Deep learning (DL) has recently emerged as a game changer within any topic related to imaging and, in particular, to medical imaging. Deep neural networks have the intrinsic capability of learning multiple abstract levels of representation. This allows for modeling complex relationships within the data, improving the overall performance of the problem to solve, either classification, estimation/regression, or reconstruction. These networks were initially proposed in the 1980s [102] although their feasibility has boosted just recently. The reason of this is the development of powerful GPUs with great processing capabilities as well as the availability of massive amounts of data.

In the field of medical image reconstruction, practitioners are very much aware of the limitations associated with the optimization-based algorithms described in previous sections. Two of them are the following: the high processing times and the need for hyperparameters tuning. Therefore, DL architectures have emerged as solutions that shift the complexity from the “production” side to the training stage. Since training is done off-line, time requirements are not an issue in this case. Of course, a number of different problems arise, and these solutions are subject to criticisms. However, it seems that this new scenario is here to stay.

Reported DL solutions can be roughly classified as those that pursue reconstruction as a black-box solution, such as [58, 103], and those that mimic the optimization process by, explicitly or implicitly, unrolling the process into several stages, for instance [55, 56]. This taxonomy is carried out in [104], and we adhere to it. However, this is a hot topic so this reference list is just a sample of recent contributions.

The field of cardiac imaging is not so populated yet. A recent contribution [105] is used (not exclusively) for static cardiac imaging. The authors propose an adversarial architecture for CS-like MRI reconstruction of static 2D images. The generator part is implemented by means of a U-Net [106]. The network is trained to learn the residuals between the fully sampled ground truth image and the zero-filling direct reconstruction. The authors highlight the importance of training a generator network as for refinement learning as well as the capability of their proposal to correctly reconstruct pathological cases despite none of them have been provided in the training stage. Static cardiac images were coherently reconstructed by a network trained with brain images, although artifacts in the blood pool region are observed as well as some loss of fine structural details.

The number of contributions related to dynamic cardiac imaging is also scarce. In [107], the authors make use of a U-Net for 2D cine reconstruction. In this case, the temporal dimension is used as an additional channel, but no further actions are accomplished to capture dynamics. Other two related contributions are [57, 104], which are described below.

The method proposed in [57] is grounded on the idea that a deep network could be trained end to end to reconstruct a dynamic sequence of cardiac images. However, it would be valuable to guarantee that the solution is coherent with the k-space information in those locations where measurements have been sampled. This leads naturally to an iterative procedure, which the authors unroll by means of a cascade of two structures, namely a deep network and a data consistency unit. The latter is a simple operation performed analytically. Both the network depth and the cascade depth are parameters to tune. The authors reshape the time sequence as a 3D volume of 2D temporal slices, so filters in the convolutional layers are spatiotemporal. In addition, they add data sharing layers as new data channels, which consist of images reconstructed by filling their subsampled k-spaces with the sampled values in nearby (in time) image frames. Despite the experiments described in the paper are preliminary, they clearly show the benefits of the proposed architecture. However, AFs are relatively low according to the state of the art described in previous sections (maximum AF is nine).

In [104], the authors avoid the network cascade by means of a recurrent architecture. This contribution runs somewhat parallel to [57]—the contribution comes from the same group—although there are a number of substantial differences. In this case, an iterative procedure based on variable splitting is used for the optimization of the overall objective function. The iteration is accomplished by means of convolutional recurrent neural networks (CRNN). In each iteration, a data consistency operation is carried out similarly to the one proposed in [57]. As for the network architecture, the authors use several layers of unidirectional CRNN as well as one layer of a bidirectional CRNN. Recurrence of unidirectional CRNN is carried out in the iterations of the optimization process. Meanwhile, the bidirectional CRNN intends to capture the dynamics of the time sequence. Consequently, recurrence in the iteration dimension and the time dimension are accounted for. Features stemming from the CRNN proposal show a higher orthogonality degree, i.e., a higher information decoupling than the features from the cascade of networks.

Overall, although this field is in its infancy, a tremendous activity is taking place in this area so amazing advances may be expected in the mid-term. However, dimensionality here is an issue. Training of 3D dynamic sequences seems tremendously involved in terms of data and computing time requirements. Maybe mixed approaches in which part of the reconstruction is carried out by means of DL solutions that are then refined by means of a classical optimization-based approach could be a procedure to explore. Time will tell.

## Data Availability

Not applicable.

## Abbreviations

2CH: 2 chambers
4CH: 4 chambers
ADMM: Alternating direction method of multipliers
AF: Acceleration factor
BH: Breath-hold
BLAST: Broad-use linear acquisition speed-up technique
CAD: Coronary artery disease
CASPR: Cartesian acquisition with spiral profile
CMRI: Cardiac magnetic resonance imaging
CRNN: Convolutional recurrent neural networks
CS: Compressed sensing
CT: Computed tomography
CVD: Cardiovascular disease
DicL: Dictionary learning
DL: Deep learning
dTV: Dynamic total variation
ECG: Electrocardiogram
FB: Free-breathing
FD: Finite difference
FFT: Fast Fourier transform
FOV: Field of view
GPU: Graphics processing unit
GRAPPA: Generalized auto-calibrating partial parallel acquisition
GW: Groupwise
GW-CS: Groupwise CS
IALM: Inexact augmented Lagrange multipliers
KLT: Karhunen-Louve transform
LGE: Late gadolinium enhancement
LLR: Locally low rank
MC: Motion compensation
MC-CS: Motion compensated CS
ME: Motion estimation
ME-MC: Motion estimation and compensation
MOCCO: Model consistency condition
MR: Magnetic resonance
MRA: MR angiography
MRI: Magnetic resonance imaging
NAV: Navigator
PC: Phase contrast
PCA: Principal component analysis
PDLDTV: Parallel DicL and dTV
PI: Parallel imaging
PSF: Partially separable function
RPCA: Robust PCA
SA: Short axis
SENSE: Sensitivity encoding
SI: Superior-inferior
SNR: Signal-to-noise ratio
SoS: Stack-of-stars
StoRM: Smoothness regularization on manifolds
tTV: Temporal total variation
ZIP: *Z* intensity-weighted position
